# Impact of Aggression on Bystanders: Quadratic Post‐Conflict Affiliation in Chimpanzees (*Pan troglodytes*)

**DOI:** 10.1002/ajp.70061

**Published:** 2025-07-11

**Authors:** Giada Cordoni, Annarita Perri, Andrea Pierdomenico, Baptiste Mulot, Ivan Norscia

**Affiliations:** ^1^ Department of Life Sciences and Systems Biology University of Torino Turin Italy; ^2^ ZooParc de Beauval & Beauval Nature Saint Aignan sur Cher France

**Keywords:** anxiety, bystander protection, quadratic affiliation, tolerance

## Abstract

In social animals, aggression is a group matter not involving only the opponents. Witnessing a conflict can induce tension and distress in bystanders (i.e., individuals not involved in either the conflict or post‐conflict affiliation with the aggressor and aggressee). For this reason, bystanders can engage in post‐conflict affiliative exchanges to reduce tension and distress, a phenomenon known as Quadratic Post‐Conflict Affiliation (QPCA). This study investigated the occurrence of QPCA in a group of chimpanzees (*Pan troglodytes*, *N* = 15) housed at ZooParc de Beauval, France. Our findings confirmed the presence of QPCA in chimpanzees under study (group QPCA tendency: 5.60% ± 2.55 SE). QPCA was primarily directed towards males, who usually tended to be more influenced by the ongoing aggression and could potentially redirect further aggression towards bystanders. High‐ranking bystanders were contacted more frequently than low‐ranking ones, as the former can potentially provide immediate protection against other aggressors and offer greater tolerance. Additionally, bystanders were less frequently targeted by aggression when QPCA was present than when it was absent. Thus, QPCA may function as a protective mechanism against aggression by other group members by reducing the chance that bystanders become victims for redirected aggression (*Bystander Protection Hypothesis*). However, QPCA failed in reducing the levels of bystanders' anxiety‐related behaviors. In conclusion, QPCA may be one of the behavioral strategies used by chimpanzees to navigate social challenges, maintain group cohesion, and mitigate aggression.

## Summary

- In chimpanzees, after a conflict, affiliation between witnesses (i.e. bystanders) may occur (Quadratic Post‐Conflict Affiliation; QPCA).
- QPCA was mostly directed toward high‐ranking bystanders by low‐ranking bystanders.
- QPCA may function as protection against aggression rather than as a calming tool for bystanders (*Bystander Protection Hypothesis*).
- QPCA occurred more often in the absence than in the presence of both reconciliation and triadic contacts (solicited and unsolicited), likely when the risk of redirection or renewed aggression is high.

## Graphical abstract

Graphical abstract representing the main results of the study.

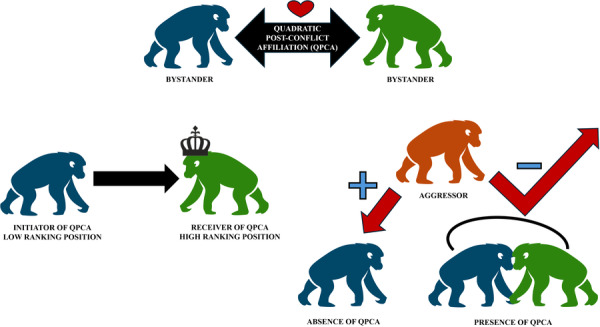

## 1 Introduction

In social animal species, including both human and nonhuman primates, conflicts—as well as tolerance and confrontation avoidance—naturally arise over resources due to the contrast between individual and group interests (*The Relational Model*; de Waal 2000). Because conflicts can spread in social groups (e.g., in human and nonhuman primates, Glomb and Liao 2003; Radford et al. 2016), balancing individual and group interests has played a crucial role in the evolution of conflict management strategies to avoid group disruption (Brandvain and Wade 2007; de Waal 2014). Besides the reunion of former opponents (reconciliation; de Waal and van Roosmalen 1979), a crucial role in the post‐conflict period is played by the witnesses that observe the conflict without being directly involved in it. Several models support the prediction that the witnesses observing a conflict may be affected by the tension that such conflict generates, as observing others in distress (such as opponents) can cause the observers to enter a state of emotional resonance and, consequently, a state of distress or agitation themselves (e.g., Preston and de Waal 2002; Decety et al. 2016; Prochazkova and Kret 2017; Yamamoto 2017). This phenomenon, also known as emotional contagion (Preston and de Waal 2002), can lead to different outcomes. Depending on the observers' level of resilience and the relationship between the observers and the distressed individuals, such emotional resonance can lead to aversion or no‐action (a phenomenon known as “bystander effect” in humans), aggressive drive (a phenomenon known as social contagion; e.g., in nonhuman primates: Videan et al. 2005; Watson and Caldwell 2010), or affiliation and prosocial behavior (e.g., Decety et al. 2016; Cutuli et al. 2018; Hortensius and De Gelder 2018; Havlik et al. 2020; Ejbye‐Ernst et al. 2022). Affiliative contacts can be effective in buffering stress and restoring group homeostasis (Cords and Thurnheer 1993; Cords and Aureli 2000; Aureli et al. 2002). After conflicts, affiliation responses can occur as triadic contacts offered by a third party not involved in the conflict to either the victim or the aggressor (Fraser and Aureli 2008; Palagi and Norscia 2013; Webb et al. 2017; Cordoni et al. 2022). These contacts can occur spontaneously (unsolicited triadic contacts) or be initiated by the victim or the aggressor approaching a third party (solicited triadic contacts) (Cordoni and Palagi 2015; Cordoni et al. 2022). A further type of post‐conflict affiliation occurs between group members not involved in the conflict as either opponents or third parties (hereafter bystanders). This type of contact is defined as Quadratic Post‐Conflict Affiliation (QPCA) (Judge and Mullen 2005; De Marco et al. 2010; Daniel and Alves 2015; Schino and Sciarretta 2015; Norscia et al. 2024).

While reconciliation and triadic contacts have been extensively investigated in various species, including humans and nonhuman primates (e.g., Butovskaya 2001; Aureli et al. 2002; Wittig and Boesch 2003; Fujisawa et al. 2005, 2006; Cordoni et al. 2016; Webb et al. 2017), relatively few studies have focused on QPCA, probably due to the operational difficulty of monitoring opponents, possible third parties and bystanders at the same time. From a functional standpoint, QPCA—as other types of affiliative interactions—may reduce anxiety (a proxy for stress; Barros and Tomaz 2002; Troisi 2002) in bystanders, thereby promoting group cohesion (Judge and Mullen 2005; Norscia et al. 2024). For example, in domestic pigs (*Sus scrofa*) QPCA reduced post‐conflict anxiety in bystanders down to baseline levels (Norscia et al. 2024). In hamadryas (*Papio hamadryas hamadryas*), bystanders increased their displacement behaviors after witnessing a conflict; however, these behaviors decreased significantly after affiliation between bystanders (Judge and Mullen 2005). In Tonkean macaques (*Macaca tonkeana*), bystanders affiliated with each other in the minutes following the conflict, thus reassuring themselves and indirectly contributing to group cohesion (De Marco et al. 2010). In mandrills (*Mandrillus sphinx*), bystanders affiliated more frequently with the victim's kin when they were related to the original aggressor (Schino and Sciarretta 2015). Also in hamadryas, the bystanders related to one combatant were more likely to affiliate with the bystanders related to the other combatant (Judge and Mullen 2005). The authors suggested that QPCA may also function as a form of “quadratic reconciliation” helping to limit the spread of aggression. Similarly, in domestic pigs levels of QPCA were lowest when preceded by reconciliation and/or triadic contacts (Norscia et al. 2024).

The levels of QPCA can be modulated by the quality of relationship between bystanders. In *Macaca fuscata*, bystanders with close relationships (i.e., kin and “friends”) exchanged more affinitive contacts after a conflict than weakly‐related bystanders (Daniel and Alves 2015). The authors discussed this result in light of relationship security and compatibility (Cords and Aureli 2000), suggesting that long‐lasting and tolerant relationships allow individuals to accurately predict their partners' actions and response in a safe context. Intriguingly, in domestic pigs QPCA mostly occurred between close kin and when either opponent was a close kin of bystanders (Norscia et al. 2024). On the other hand, in hamadryas, QPCA occurred more frequently between non‐kin compared to kin (Judge and Mullen 2005). However, the non‐kin with which bystanders interacted were preferred social partners.

Here, we investigated the occurrence and potential modulation of QPCA in chimpanzees (*Pan troglodytes*). Chimpanzees live in a multi‐male/multi‐female fission–fusion society characterized by male philopatry and mainly based on long‐lasting and cooperative social bonds between males (Gilby and Wrangham 2008; Bray and Gilby 2020; Bray et al. 2021). In contrast, females do not generally form strong social relationships, even though their relations can vary across different populations and socio‐ecological conditions (Thompson et al. 2007; Wakefield 2008, 2013). As a matter of fact, it has been suggested that in the wild male–female and female–female dyads may constitute valuable relationships as do male–male dyads (Wittig and Boesch 2003; Lehmann and Boesch 2009). In captivity, females are often closely related and motivated to exchange valuable services such as grooming and agonistic support (Fraser et al. 2008).

Aggression is contagious in chimpanzees and can spread within the group and across different groups (Baker and Aureli 1996; Videan et al. 2005; Palagi et al. 2006). Moreover, reconciliation and triadic solicited and unsolicited contacts—including consolation—have already been documented in both captive and wild chimpanzees (de Waal and van Roosmalen 1979; Arnold and Whiten 2001; Wittig and Boesch 2003; Kutsukake and Castles 2004; Palagi et al. 2006; Koski et al. 2007; Fraser and Aureli 2008; Fraser et al. 2008; Romero et al. 2010; Webb et al. 2017). Thus, aggressive events in chimpanzees are known to impact the behavior of all group members. However, to date, no data are available in the literature regarding the presence of QPCA in chimpanzees. Therefore, we investigated this issue by testing the following predictions.

Prediction 1 Presence of Quadratic Post‐Conflict Affiliation (QPCA)

Based on evidence of the involvement of group members other than the opponents during post‐conflict periods, we expected that QPCA would occur between bystanders in chimpanzees, as observed in other species (Judge and Mullen 2005; Leone et al. 2010; De Marco et al. 2010; Daniel and Alves 2015; Schino and Sciarretta 2015; Norscia et al. 2024).

Prediction 2 Individual and social factors linked to bystanders modulating QPCA

The chimpanzee society is defined as a “male‐bonded society” and males are the dominant sex (Goodall 1986; Boesch 2009; Gruber and Clay 2016). Based on this, we expected that QPCA would be directed more towards males than females (*Prediction 2a*). According to the *Valuable Relationship Hypothesis* (de Waal and Aureli 1997; Wittig and Boesch 2003), post‐conflict mechanisms are more likely to occur between individuals who share strong bonds (e.g., kin) or social relationships that provide benefits such as protection by dominant individuals. In this view, we expected that QPCA would be directed more frequently towards kin than non‐kin and high‐ranking rather than low‐ranking individuals (*Prediction 2b*).

Prediction 3 Individual and social factors linked to the opponents and aggression features modulating QPCA

In hamadryas, bystander responses may depend on both the identity of the previous opponents and their relationships with bystanders (Judge and Mullen 2005). In contrast, in mandrills QPCA was not modulated by either the characteristics of the preceding aggressive event (e.g., intensity, unidirectionality/bidirectionality) or the original opponents (Schino and Sciarretta 2015). Based on chimpanzee social dynamics, we expected that QPCA would occur more frequently when the aggressor was a high‐ranking male (the dominant sex) due to the potential risk of renewed aggression from them (*Prediction 3a*). Additionally, since third party post‐conflict contacts in chimpanzees may be more common following severe aggression (Palagi et al. 2006), we expected that QPCA would occur after high‐intensity conflicts (*Prediction 3b*).

Prediction 4 QPCA, anxiety reduction and renewed aggression

Aggression is one of the major sources of anxiety for social groups (Aureli et al. 2002). Specific behaviors can be associated with the increasing anxiety in many mammal species, such as self‐scratching, yawning, vacuum behavior, and head/body shaking (Fried 1994; Bögels and Reith 1999; Troisi 2002; Norscia et al. 2021). In the wild, chimpanzee females scratched more frequently when in proximity to less compatible partners (Kutsukake 2003). In captivity, chimpanzees increase their scratching and yawning behaviors in response to vocalizations from neighboring groups (Baker and Aureli 1996), crowding conditions (Aureli and De Waal 1997), and other socially arousing situations (Hopkins et al. 2006). Affiliation between bystanders may function in reducing anxiety generated by the conflict (Judge and Mullen 2005; Daniel and Alves 2015). In this light, we expected that, in chimpanzees, anxiety levels of bystanders may be higher before rather than after the QPCA (*Prediction 4a*). Quadratic affiliation may also limit the probability of renewed aggression towards bystanders, as it has been observed in hamadryads and Japanese macaques (Daniel and Alves 2015). For this reason, we expected that the presence rather than the absence of QPCA would reduce the levels of renewed aggression towards bystanders (*Prediction 4b*).

Prediction 5 Relation between QPCA and other post‐conflict mechanisms

In chimpanzees, affiliation directed towards the aggressee by a bystander can be considered an alternative mechanism used to alleviate aggressee anxiety and limit the probability of renewed aggression within the group (Wittig and Boesch 2003; Palagi et al. 2006; Judge and Bachmann 2013). If QPCA functions to reduce bystander anxiety and the risk of attacks that bystanders might face (see *Prediction 4*), we expected that QPCA would occur more before rather than after reconciliation and triadic solicited/unsolicited contacts.

## 2 Methods

### 2.1 Ethics Statement

The current study was purely observational and non‐manipulative; thus, approval was not required by the authors' institutional animal care committees. The study adhered to the American Society of Primatologists (ASP) Principles for the Ethical Treatment of Nonhuman Primates and to the legal requirements of the country in which the research was conducted.

#### 2.1.1 The Study Group

The current study was conducted on the chimpanzee colony (*Pan troglodytes*) housed at the ZooParc de Beauval and composed of 15 individuals (see Table 1). Chimpanzees were in *Continuous Full Contact*. Six females of the group (Baraka, Bonobo, Charlotte, Julie, Sangha, and Wamba) were treated with oral contraceptives and two males (Lukombé and Tumba) were vasectomized. Animals occupied an indoor facility of about 300 m^2^ and an island surrounded by a moat of 3000 m^2^. Both facilities were equipped with platforms, ropes, trunks, hammocks, straw and vegetation. Environmental enrichments such as artificial termite nest and task maze were also provided. The animal received food (i.e., vegetables, seed/pellet cake, fruits, and meat) six times per day. Water was available *ab libitum*.

**TABLE 1:** The chimpanzee group under study housed at the ZooParc de Beauval (France). The number of attracted, dispersed, and neutral pairs for each individual was reported.

Subject	Age* (years)	Sex	Mother‐offspring	Attracted pairs	Dispersed pairs	Neutral pairs
BARAKA	44	F	*	6	4	17
BONOBO	41	F	Mother of Sangha, Wamba, Lukombé, Yumbi	4	2	20
CHARLOTTE	47	F	Mother of Domi, grandmother of Lobai, N'saka, Tumba	1	3	29
DOMI	34	F	Mother of Lobai, N'saka, Tumba	4	0	28
GYPSO	36	F	*	0	6	23
JULIE	41	F	*	8	3	27
MICHELINE	33	F	*	5	0	23
SANGHA	17	F	*	2	0	28
WAMBA	15	F	*	3	4	33
JOSEPH	48	M	*	2	2	23
LUKOMBÈ	12	M	*	6	5	9
TUMBA	9	M	*	10	7	17
LOBAI	9	M	*	4	1	23
YUMBI	9	M	*	9	5	9
N'SAKA	2	M	*	8	6	21

* Individual ages during the period of observation.

#### 2.1.2 Data Collection and Operational Definitions

Video and live data were collected from April to August 2023 by two observers (A.Pe. and A.Pi.). Observations were carried out daily, alternating morning (09:00–12:00) and afternoon (14:00–19:00), both indoor and outdoor. Videos were recorded via Full HD, via Panasonic HC‐V380/V180 and Sony HDR‐PJ240E cameras. The aggression and post‐aggression periods were filmed. The camera operators focused on the opponents and surrounding individuals, maintaining a wide‐angle frame to capture as many individuals as possible; however, not all 15 individuals were always in view. However, at least four surrounding individuals were always present in the videos (mean ± SD 7.04 ± 2.5, minimum and maximum values 4.0 and 14.0). G.C. and I.N. trained both observers for 30 h. in chimpanzee identification, data collection methods, and behavioral pattern distinction (Table 2). Training ended when interobserver availability rate—measured by Cohen's K—reached a value of 1.00 for animal identification and 0.81 for behavioral pattern distinction. Video analysis was made by using Avidemux 2.7.1 and PotPlayer, often frame‐by‐frame or in slow motion. We collected a total of 150 h. of observations.

**TABLE 2:** Behavioral patterns recorded in the chimpanzee group under study.

Behavioral patterns	Description
Affiliative behaviors	
Body contact	Two or more chimpanzees are lying down, sitting, or standing with a part (or more parts) of their bodies in contact
Embrace	A chimpanzee wraps their arm around the shoulder or back of a companion or two chimpanzees approach each other and initiate contact by wrapping their arms around the other's body and placing their heads at the other's shoulder or abdomen
Grooming	A chimpanzee manipulates and cleans the fur of a companion with their hands or mouth
Kiss	A chimpanzee opens their mouth and places it on a part of the companion's body without closing it
Aggressive, fear, and defensive behaviors	
Aggressive bite	A chimpanzee closes their mouth on the companion's body
Aggressive jump	A chimpanzee jumps on the opponent only with feet generally in a quite bipedal position
Aggressive pull	A chimpanzee moves the opponent towards them with hands and feet
Aggressive push	A chimpanzee displaces the opponent far from them with hands/feet. They can perform a push for defending themselves from the attack of the opponent or for attacking the opponent
Aggressive retrieve	A chimpanzee blocks the opponent with their hands to prevent them moving away. It is different from pull, which is generally performed with both feet and hands
Aggressive slap	A chimpanzee hits with the palm of their open hand any part of the opponent's body
Aggressive stamp	A chimpanzee hits the ground or the opponent with their feet in a repeated way
Avoid	A chimpanzee moves away from the path when another individual is approaching them or takes a less direct route around the other
Bared‐teeth	A chimpanzee's mouth corners are withdrawn and the lips retracted from teeth and gums. The mouth can be kept closed or slightly opened. It can be associated with screaming
Charging display	A chimpanzee performs specific postures, movements, piloerection, facial expressions, and vocalizations for threatening the opponent
Crawl/Crouch	A chimpanzee bends all four limbs, presses their ventrum to the ground, and tries to travel while in this position or crouches while sitting by lowering the head, hunching the shoulders, and often covering the head with their arm/s
Flee	A chimpanzee runs away from the partner that runs behind them. While running away, the chimpanzee often changes their direction, sometimes stops and looks back to check for the presence of the partner
Scream	A chimpanzee emits a high‐pitched, high‐volume frightened vocalization
Shelter	A chimpanzee protects themselves from the attack of the opponent by putting her/his arms over the head
Wriggle	A chimpanzee moves to get rid of the grip of the opponent
Submissive behaviors	
Bob	A chimpanzee bends their back and weaves with head or whole body in a bowing position upwards or forward (Roberts et al. 2014). This is a typical submissive behavior that can be performed by subordinates to avoid being attacked
Hold out hand or Wrist Present (Fultz et al. 2022)	A chimpanzee extends their arm and presents an outstretched bent wrist as a submissive gesture to a dominant individual
Pant‐grunt	It is an acoustically heterogeneous signal usually consisting of repeated grunts that can be panted and graded into barks or screams
Present	Subordinate chimpanzee shows hindquarters to a dominant companion in nonsexual manner
Anxiety‐related behaviors	
Scratching	A chimpanzee uses their hands/feet to rub part of their body
Self‐grooming	A chimpanzee uses their hands and/or teeth to clean their body
Yawning	A chimpanzee performs deep, long inhalation with open mouth

We evaluated by live coding the levels of whole group activity (i.e., moving, resting, feeding, proximity, body contacting, grooming, playing, and aggression) by using the scan animal sampling method (Altmann 1974) at 10‐min intervals.

**FIGURE 1:**
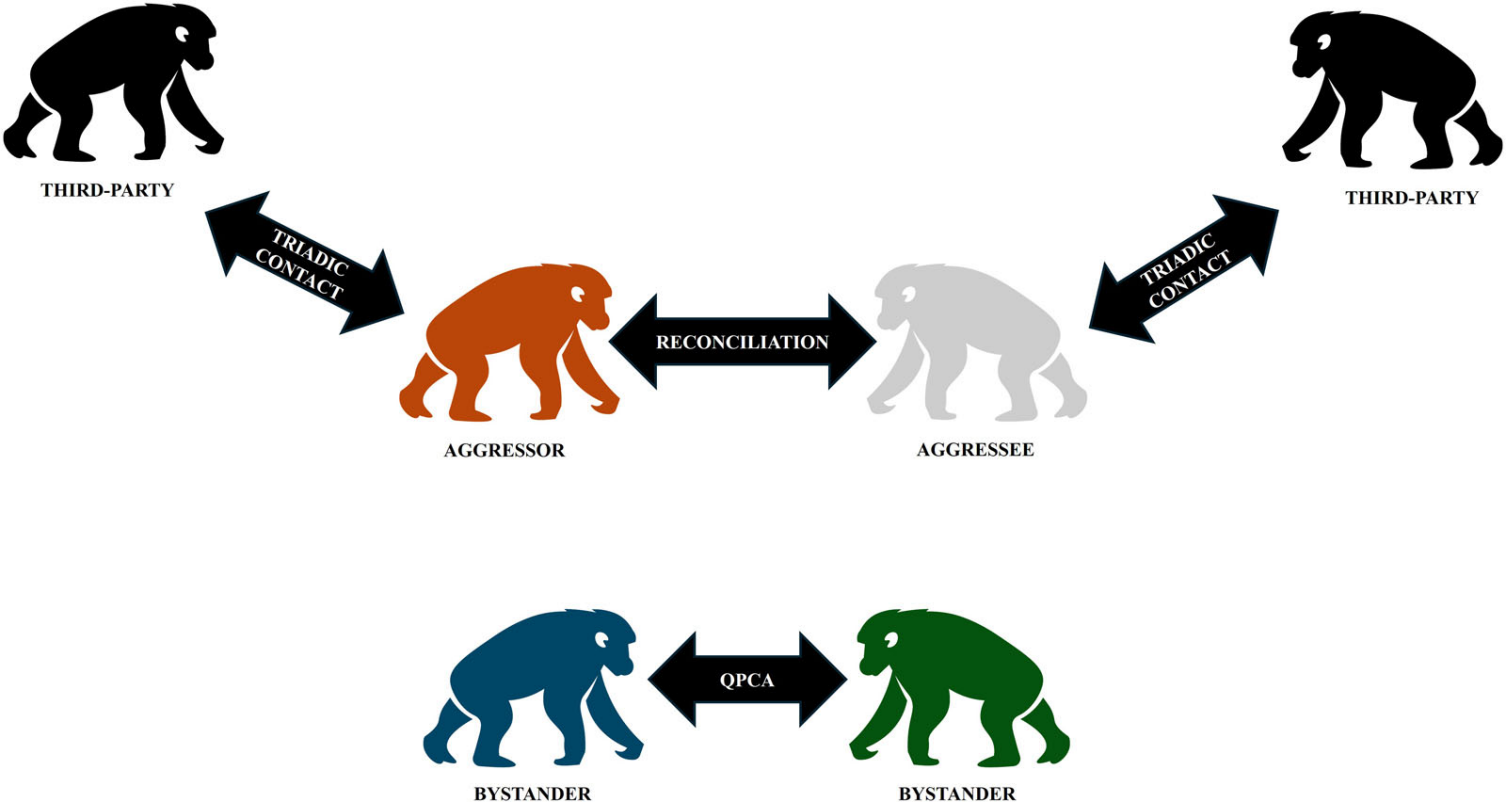
Figure representing the different post‐conflict mechanisms: reconciliation between the two chimpanzees directly engaging in the aggression (i.e., aggressor and aggressee), solicited and unsolicited triadic contacts between chimpanzees not involved in the conflict (i.e., third parties) with aggressor and/or aggressee, and quadratic post‐conflict affiliation (QPCA) between chimpanzees witnessing the aggression but not involved in the conflict and in any post‐conflict affiliation with either opponent (i.e., bystanders).

For the animals in view, all occurrences sampling method (Altmann 1974) was used to video record all aggressive events, post‐aggressive interactions and anxiety‐related behaviors (i.e., scratching, self‐grooming, and yawning; Maestripieri et al. 1992; Baker and Aureli 1997; Guggisberg et al. 2010; Table 2). For each aggressive event we determined: (i) intensity, distinguishing between aggression with physical contact and/or fearing vocalizations (e.g. screaming) between opponents (high‐intensity aggression) and aggression without physical contact and/or fearing vocalizations (low‐intensity aggression), (ii) which of the two opponents was the winner (i.e. the individual who attacked and/or pursued the opponent without displaying any fear/submissive behaviors) or the loser (i.e. the individual who defended themselves and/or flew away from the partner attack and performed fear/submissive behavioral patterns, Table 2; Cordoni et al. 2023) of the aggressive encounter—in case of decided aggression the winner and loser were clearly distinguished (we did not record any decided aggression during which the winner screamed while chasing the opponent), whereas in case of undecided aggression, they were not; and (iii) the duration in seconds. As in Norscia et al. (2024), after an aggressive event we distinguished (i) opponents—the two chimpanzees directly engaging in the aggression, (ii) third parties—chimpanzees not involved in the conflict that engaged in unsolicited or solicited affiliative contacts with one (or both) of the former opponents after the conflict, and (iii) uninvolved bystanders (hereafter, bystanders)—chimpanzees witnessing the aggression but not involved in the conflict and in any post‐conflict affiliation with either opponent (Figure 1).

We employed the PC‐MC method, which is commonly used to study reconciliation, triadic contacts, and QPCA (de Waal and Yoshihara 1983; Judge and Mullen 2005; Arnold and Aureli 2007). Following each aggressive event, all bystanders visible in the video were monitored for a 3‐min post‐conflict period (PC). The number and identities of bystanders varied randomly depending on the area captured in the video frame. For this reason, we tracked individuals rather than dyads, as it was not possible to know in advance if and which bystanders might engage in affiliative interactions. On average, each individual was observed as a bystander in 89 ± 19.6 SD post‐conflict events. A 3‐min time window was employed as it has been shown that in primates post‐conflict interactions mainly occurred within the first two/3 min (Preuschoft et al. 2002; Palagi et al. 2006; Judge and Mullen 2005; Cordoni et al. 2006; Leone et al. 2010). Each PC observation was paired with a 3‐min matched‐control observation (MC) conducted on the next possible day in the same social (i.e., at least four individuals present to provide the bystander with a similar opportunity for social interactions as in PC; Cordoni et al. 2022) and environmental context (same enclosures, weather, and time ± 30 min) on original bystanders, with no aggression occurring in the preceding 10 min. In MC, bystanders of the previous PC may affiliate with any individuals of the group to ensure that the probability of interactions in the control condition is the same as in the post‐conflict condition. We then classified PC–MC pairs as: (i) attracted (AP) when the first affiliative contact between bystanders occurred earlier in PC than MC or only in PC), (ii) dispersed (DP) when the first affiliative contact occurred earlier in MC than PC or only in MC), and (iii) neutral (NP) when the first affiliative contact occurred in both PC and MC at the same minute or in neither condition. We collected a total of 450 PC–MC pairs. The presence of QPCA can be confirmed only if the number of APs is significantly higher than the number of DPs (de Waal and Yoshihara 1983). If QPCA is demonstrated, individual QPCA tendency can be calculated using the following formula adapted from Veenema et al. (1994): (APs − DPs)/(APs + DPs + NPs) * 100.

We then evaluated the potential influence of individual (i.e., sex) and social factors (i.e., kinship, relationship quality, and rank) on the levels of QPCA (number of quadratic affinitive contacts initiated by an individual/number of times that individual was a bystander). In the different analyses, QPCA levels were normalized based on the number of males/females, kin/non‐kin, and higher‐ranking/lower‐ranking individuals present in the group. Since the proportion of these categories (i.e., males/females, kin/non‐kin, and higher‐ranking/lower‐ranking individuals) of partners available in each PC was not the same as those in the group, this observation technique can be fine‐tuned in further analyses to account for this limitation.

To measure the relationship quality, we calculated the hourly frequency of grooming plus body contact for each dyad. Furthermore, we calculated the median value of all dyadic affiliation frequencies and evaluated whether each dyad had an affiliation frequency above or below the median. We considered as kin mother‐offspring dyads and siblings.

To assess the potential anxiolytic effect of QPCA, we calculated the frequency per minute of anxiety‐related behaviors exhibited by bystanders across four different periods in absence of reconciliation: (i) control period (MC), not preceded by any aggressive event in the previous 10 min (this is the same period as in the PC–MC method), (ii) pre‐aggression period (PRE), the 3 min before the start of the aggression, (iii) post‐conflict period with any QPCA (PC_NO_), and (iv) post‐conflict period after the occurrence of QPCA (PC_YES_).

#### 2.1.3 Statistical Analysis

All distributions deviated from normality (Kolmogorov‐Smirnov test *p* < 0.001); therefore, we employed non‐parametric statistics (Hoskin 2012). Specifically, we used the Kruskal–Wallis test for comparing k‐independent samples (e.g., hourly frequency of aggression across the diverse sex‐class combinations) with 10,000 randomizations. In case of significance, a post hoc test was applied for pairwise comparison. For comparing k‐dependent samples (e.g., frequency of anxiety‐related behaviors across four periods), we used the Friedman test (10,000 permutations) followed—if significant—by post hoc test for pairwise comparisons. We applied the Wilcoxon signed‐rank exact test for comparing two dependent samples, such as number of attracted pairs versus dispersed pairs. Furthermore, we also used the Wilcoxon test to compare the number of times each bystander affiliated with another bystander with whom they shared either a close (above the median value of all dyadic affiliation hourly frequencies) or a weak (below the median) social relationship. Finally, we used the Spearman correlation test to assess potential correlations between the frequency of aggression and rank values, as well as between the rank values of both initiator and receiver of quadratic affinitive contacts.

We ran three GLMMs to assess the potential influence of both opponent and aggression features on the presence of QPCA. All GLMMs (*N*_events_ = 460) included as target variable the occurrence of QPCA (binomial variable: presence = 1, absence = 0) and as random factors the aggressor, aggressee, and initiator of the QPCA identities. The first model (GLMM_individual_factors_) included as fixed factors the sex of both the aggressor and the aggressee (binomial variable: male = 0, female = 1). The second model (GLMM_social_factors_) included as fixed factors the Normalized David's Score (NDS) values of the aggressor, aggressee, and initiator of the QPCA (scale variable), the relationship quality between aggressor and initiator and aggressee and initiator (scale variable), and the kinship between aggressor and initiator and aggressee and initiator (binomial variable: kin = 1, non‐kin = 0). The last model (GLMM_aggression_features_) included as fixed factors the aggression intensity (binomial variable: high‐intensity = 1, low‐intensity = 0), identification of the winner and loser (binomial variable: decided = 1, undecided = 0), and aggression duration (scale variable).

To evaluate individual ranking positions, we calculated for each chimpanzee the NDS on the basis of the observed outcomes of dyadic aggression (number of conflicts won or lost by each subject; de Vries 1993; de Vries et al. 2006). In the calculation of NDS, the observed proportion of wins was corrected for the chance occurrence of the observed outcome based on a binomial distribution with each subject having an equal chance to win or lose in every conflict. The correction is necessary when, as in the case of our study, the number of aggressive events differed between pairs.

## 3 Results

### 3.1 Preliminary Results

The hourly frequency of aggression significantly differed across the diverse sex‐class combinations (Kruskal–Wallis test *N*_MMdyads_ = 30, *N*_MFdyads_ = 54, *N*_FMdyads_ = 54, *N*_FFdyads_ = 72, *H* = 52.096, df = 3, *p* < 0.001). Post hoc test revealed the following results: MM versus MF (*Q* = −0.887, *p* = 1.000), MM versus FM (*Q* = 39.094, *p* = 0.011), MM versus FF (*Q* = 62.199, *p* < 0.001), MF versus FM (*Q* = 39.981, *p* = 0.001), MF vs. FF (*Q* = 63.086, *p* = 0.001), and FM vs. FF (*Q* = 23.104, *p* = 0.117). Thus, males performed more attacks as aggressors than females.

Prediction 1 Presence of Quadratic Post‐Conflict Affiliation (QPCA)

**FIGURE 2:**
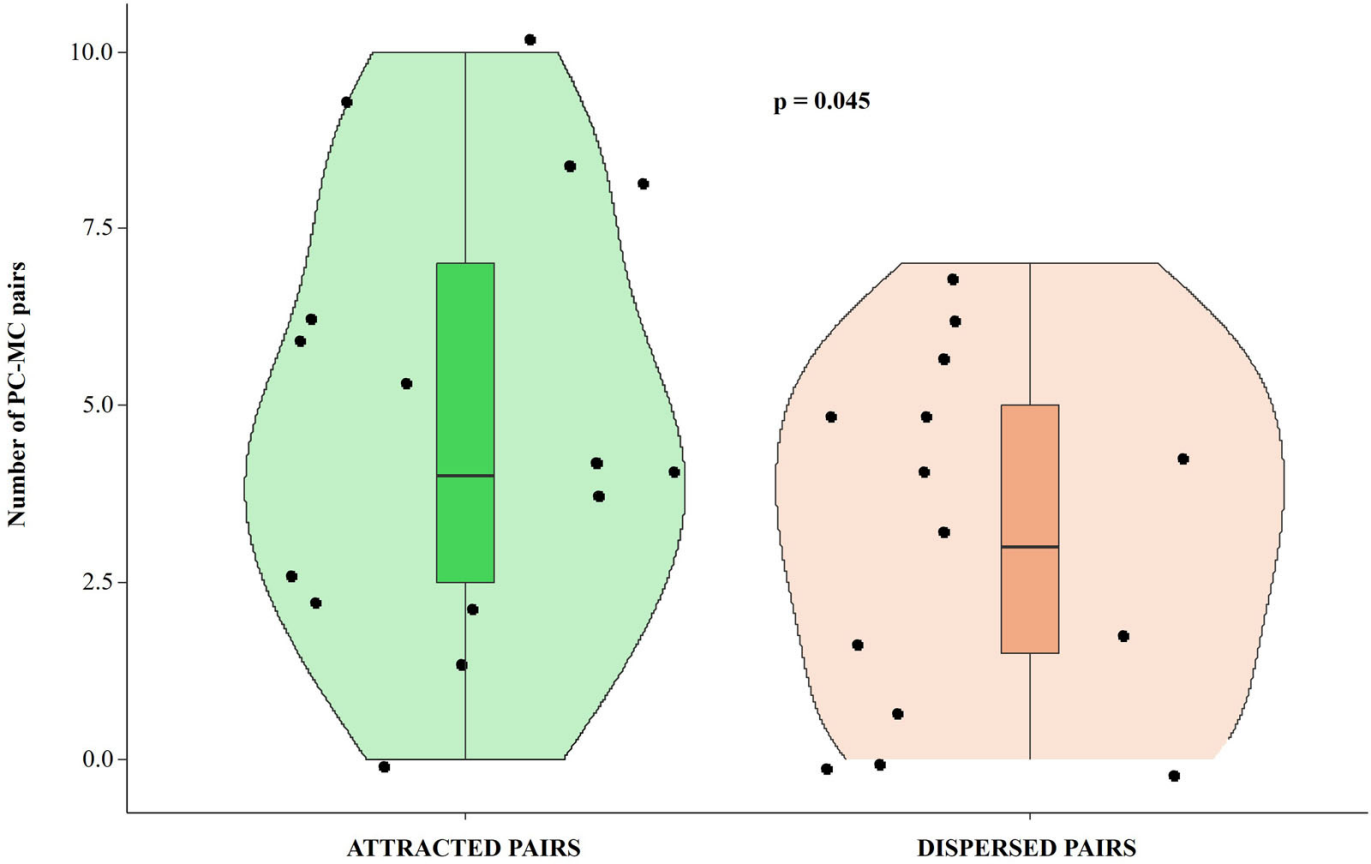
Violin plots with included boxplots representing the number of attracted and dispersed pairs for the analysis of the occurrence of quadratic post‐conflict affiliation. Shape of the violin represents the density estimate of the variable considered: the more data points in a specific range, the larger the violin shape is for that range. Dots represent the individual data points (*N*_individuals_ = 15). In the middle of each density curve, there is a small boxplot with the rectangle showing the ends of the first and third quartiles and the central line showing the median value.

We found that the attracted pairs were significantly higher than dispersed pairs (Wilcoxon exact test *N*_individuals_ = 15, T = 20.5, ties = 1, *p* = 0.045; Figure 2). Hence, the phenomenon of post‐conflict quadratic affiliative contacts was present in the chimpanzee group under study. The mean and median values of QPCA tendency were 5.60% ± 2.55 SE and 7.41%, respectively.

Prediction 2 Individual and social factors modulating QPCA

**Prediction *2*a**. The individual initiating quadratic affiliation directed their contact more frequently towards males than females (Wilcoxon exact test *N*_individuals_ = 15, *T* = 11.0, ties = 0, *p* = 0.003).

**FIGURE 3:**
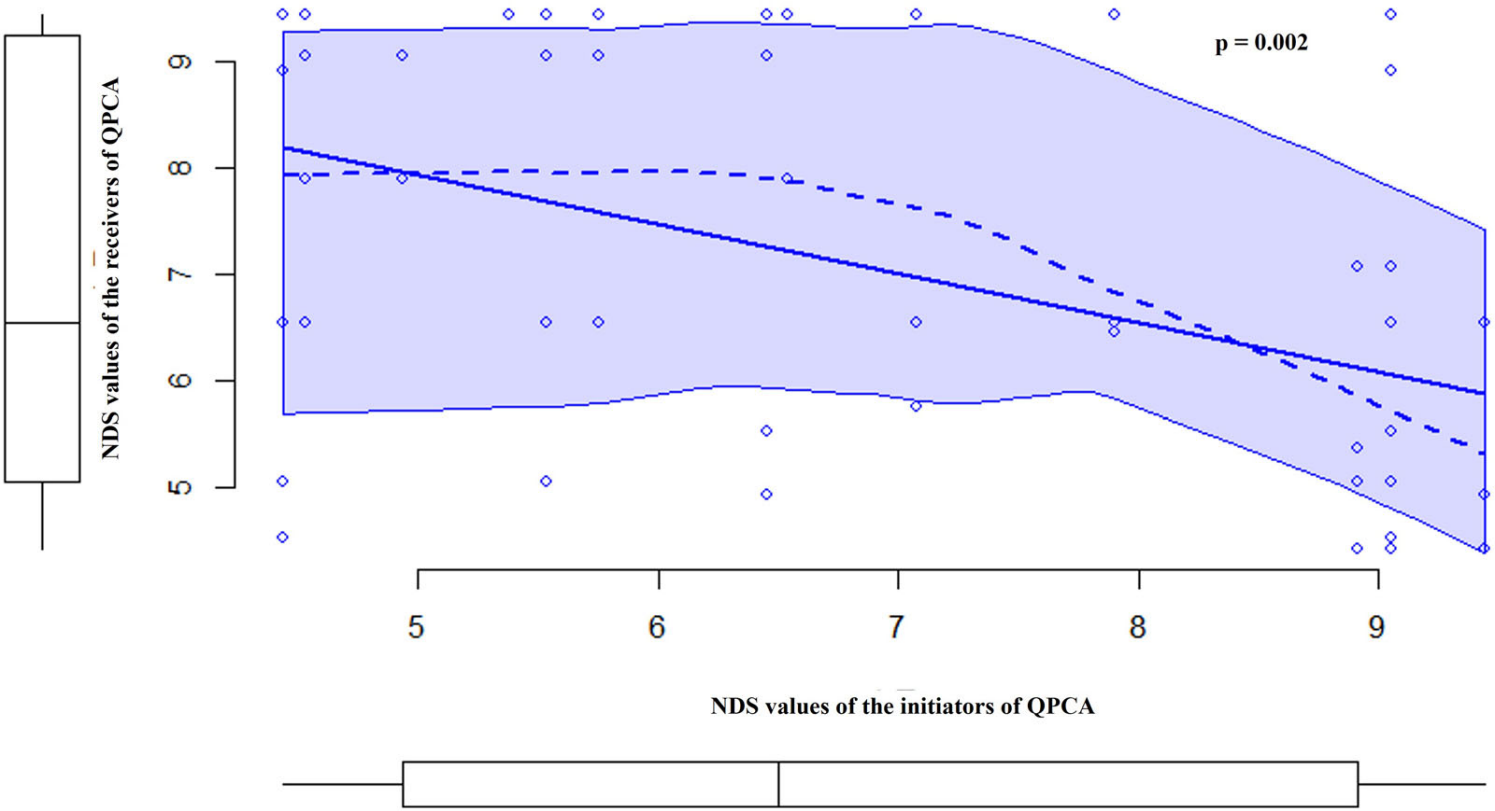
Scatterplot with marginal boxplots representing the negative correlation between the Normalized David's Score (NDS) values (the higher the NDS value, the higher the individual ranking position) of the receivers of the quadratic post‐conflict affiliation (QPCA; *Y*‐axis) and the NDS values of the initiators of QPCA (*X*‐axis). Solid blue line: the regression line (it is added by default by the scatterplot function in R even if the distribution is not parametric). Dashed blue line: the non‐parametric regression smooth. Light blue area: the smooth conditional spread. Blue dots: representing each chimpanzee dyad. In each marginal boxplot, median, maximum and minimum values, and quartiles of distribution are represented.

**Prediction *2*b**. Neither kinship (Wilcoxon exact test *N*_individuals_ = 10, *T* = 23, ties = 0, *p* = 0.695; only chimpanzees having kin in the group were included in the analysis of kinship) nor relationship quality (Wilcoxon exact test *N*_individuals_ = 15, *T* = 35.0, ties = 3, *p* = 0.791) affected the levels of quadratic affiliations. However, the ranking positions of individuals initiating quadratic affiliations negatively correlated with the ranking positions of individuals receiving quadratic affiliations (Spearman correlation test *N*_dyads_ = 60, *r_S_* = −0.399, *p* = 0.002; Figure 3). Furthermore, individuals initiating quadratic affiliations directed their contacts more towards high ranking rather than low ranking individuals (Wilcoxon exact test *N*_individuals_ = 11, *T* = 0, ties = 0, *p* < 0.001; median levels of quadratic affiliation and interquartile range: towards high‐ranking individuals 0.0274/0.02; towards low‐ranking individuals 0.000/0.02). We excluded from this analysis the infant (N'saka), Gypso (she did not perform any quadratic affiliative contacts), and Lukombé and Julie, covering the highest and the lowest ranking position, respectively.

Prediction 3 Individual and social factors linked to the opponents and aggression features modulating QPCA

**Prediction *3*a**. Considering the models GLMM_individual_factors_ and GLMM_social_factors_, full models did not differ from the null models (GLMM_individual_factors_ ‐ likelihood ratio test: *χ* ^2^ = 5.467, df = 2, *p* = 0.065; GLMM_social_factors_ ‐ likelihood ratio test: *χ* ^2^ = 5.868, df = 7, *p* = 0.555; see Table 3). Thus, neither individual (i.e., sex of aggressor and aggressee) nor social (i.e., NDS values of the aggressor, aggressee, and initiator of QPCA, relationship quality between aggressor and initiator and aggressee and initiator, and kinship between aggressor and initiator and aggressee and initiator) factors linked to the original opponents affected the presence/absence of QPCA.

**Prediction *3*b**. Considering the aggression features (i.e., aggression intensity, identification of the winner and loser, and aggression duration), the full model did not differ from the null model (likelihood ratio test: *χ* ^2^ = 4.509, df = 3, *p* = 0.211; see Table 3). Thus, none of the aggression characteristics considered influenced the occurrence of QPCA.

**TABLE 3:** Results of the generalized linear mixed models (GLMMs).

	Estimate	ES	*z*‐Value	*p*
GLMM_individual_factors_ (*N*_events_ = 460) target variable: presence/absence of QPCA random factors: identities of aggressor, aggressee, and the initiator of the QPCA full model vs. null model: *χ* ^2^ = 5.467, df = 2, *p* = 0.065				
Intercept	−2.092	0.424	−4.938	< 0.001
Sex aggressor	−1.221	1.070	−1.141	0.254
Sex aggressee	0.739	0.445	1.662	0.097
GLMM_social_factors_ (*N*_events_ = 460) target variable: presence/absence of QPCA random factors: identities of aggressor, aggressee, and the initiator of the QPCA full model versus null model: *χ* ^2^ = 5.868, df = 7, *p* = 0.555				
Intercept	−0.366	1.410	−0.260	0.795
NDS aggressee	−0.049	0.163	−0.298	0.766
NDS aggressor	−0.213	0.131	−1.629	0.103
NDS initiator	0.103	0.079	1.308	0.191
Bond aggressee‐initiator	4.102	5.520	0.743	0.457
Bond aggressor‐initiator	0.898	2.579	0.348	0.728
Kinship aggressee‐initiator	0.382	0.375	1.018	0.309
Kinship aggressor‐initiator	−0.002	0.318	−0.005	0.996
GLMM_aggression_features_ (*N*_events_ = 460) target variable: presence/absence of QPCA random factors: identities of aggressor, aggressee, and the initiator of the QPCA full model versus null model: *χ*2 = 4.509, df = 3, *p* = 0.211				
Intercept	−2.526	0.561	−4.501	< 0.001
Aggression duration	0.005	0.036	0.141	0.888
Aggression intensity	0.478	0.402	1.190	0.234
Aggression decided‐undecided	0.647	0.441	1.466	0.143

Prediction 4 QPCA, anxiety reduction, and renewed aggression

**FIGURE 4:**
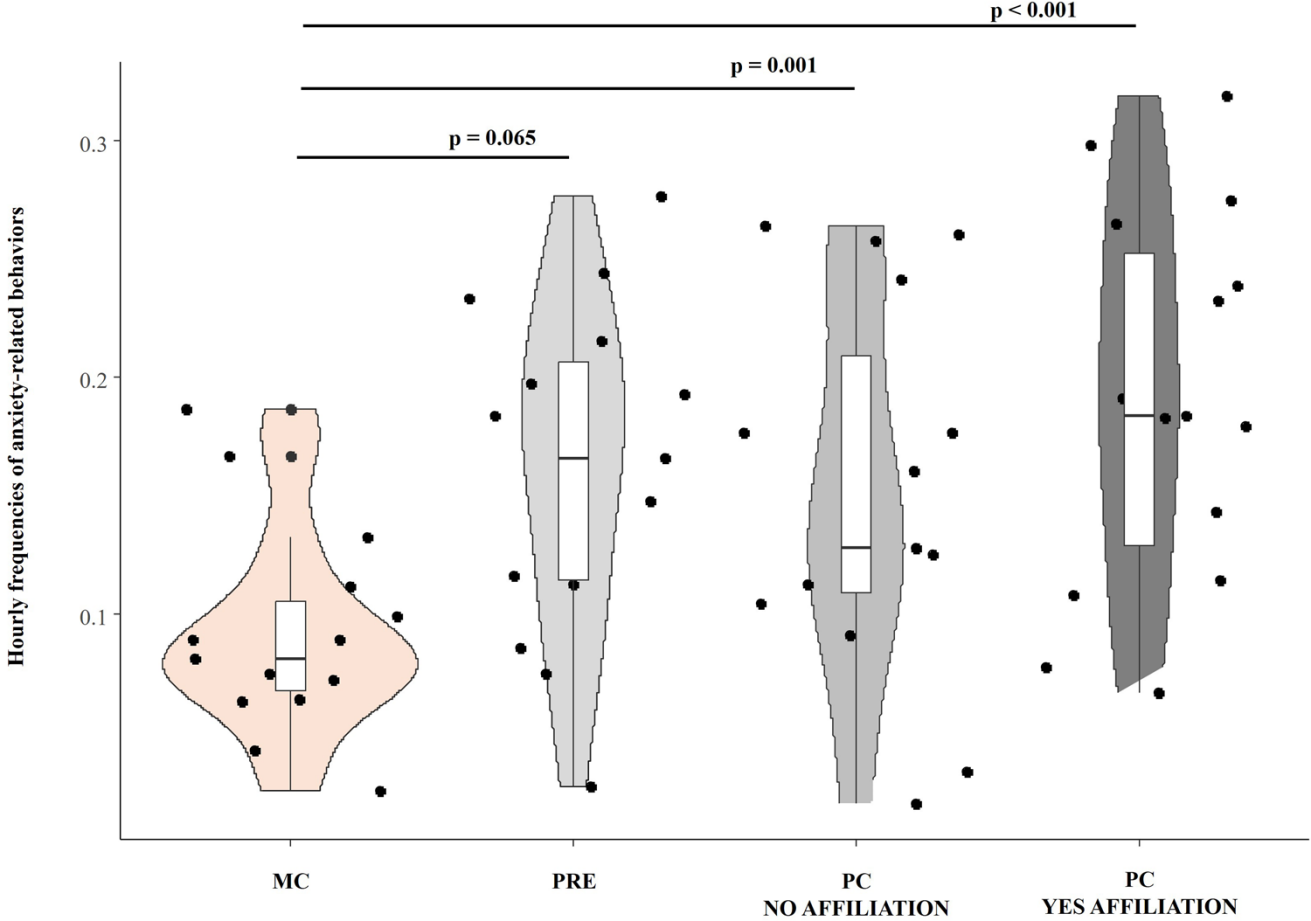
Violin plots with included boxplots representing the hourly frequency of anxiety‐related behaviors under four conditions: (i) control period (MC), not preceded by any aggressive event in the previous 10 min, (ii) pre‐aggression period (PRE), the 3 min before the start of the aggression, (iii) post‐conflict period with any QPCA (PC NO AFFILIATION), and (iv) post‐conflict period after the occurrence of QPCA (PC YES AFFILIATION). Shape of the violin represents the density estimate of the variable considered: the more data points in a specific range, the larger the violin shape is for that range. Dots represent the individual data points (*N*_individuals_ = 15). In the middle of each density curve, there is a small boxplot with the rectangle showing the ends of the first and third quartiles and the central line showing the median value.

**Prediction *4*a**. We compared the levels of anxiety‐related behaviors of bystanders across the four conditions (MC, PRE, PC_NO_, PC_YES_) and we found a significant difference (Friedman exact test *N*_individuals_ = 15, χ2 = 21, df = 3, *p* < 0.001; median values and interquartile range: MC 0.075/0.03, PRE 0.148/0.10, PC_NO_ 0.127/0.17, PC_YES_ 0.180/0.12; Figure 4). In particular, the post hoc test (Wilcoxon signed‐rank tests, Bonferroni's correction applied) results were: MC vs. PRE (*q* = −1.200, *p* = 0.065), MC vs. PC_NO_ (*q* = −1.800, *p* = 0.001), MC versus PC_YES_ (*q* = −1.933, *p* < 0.001), PRE versus PC_NO_ (*q* = −0.600, *p* = 1.000), PRE versus PC_YES_ (*q* = −0.073, *p* = 0.719), and PC_NO_ versus PC_YES_ (*q* = −0.133, *p* = 1.000). Accordingly, lower levels of anxiety‐related behaviors were observed only in the control condition (i.e., when no prior aggression had occurred), compared to the other conditions (PRE, PCYES, and PCNO). Thus, post‐conflict quadratic affiliation did not alleviate anxiety in bystanders.

**FIGURE 5:**
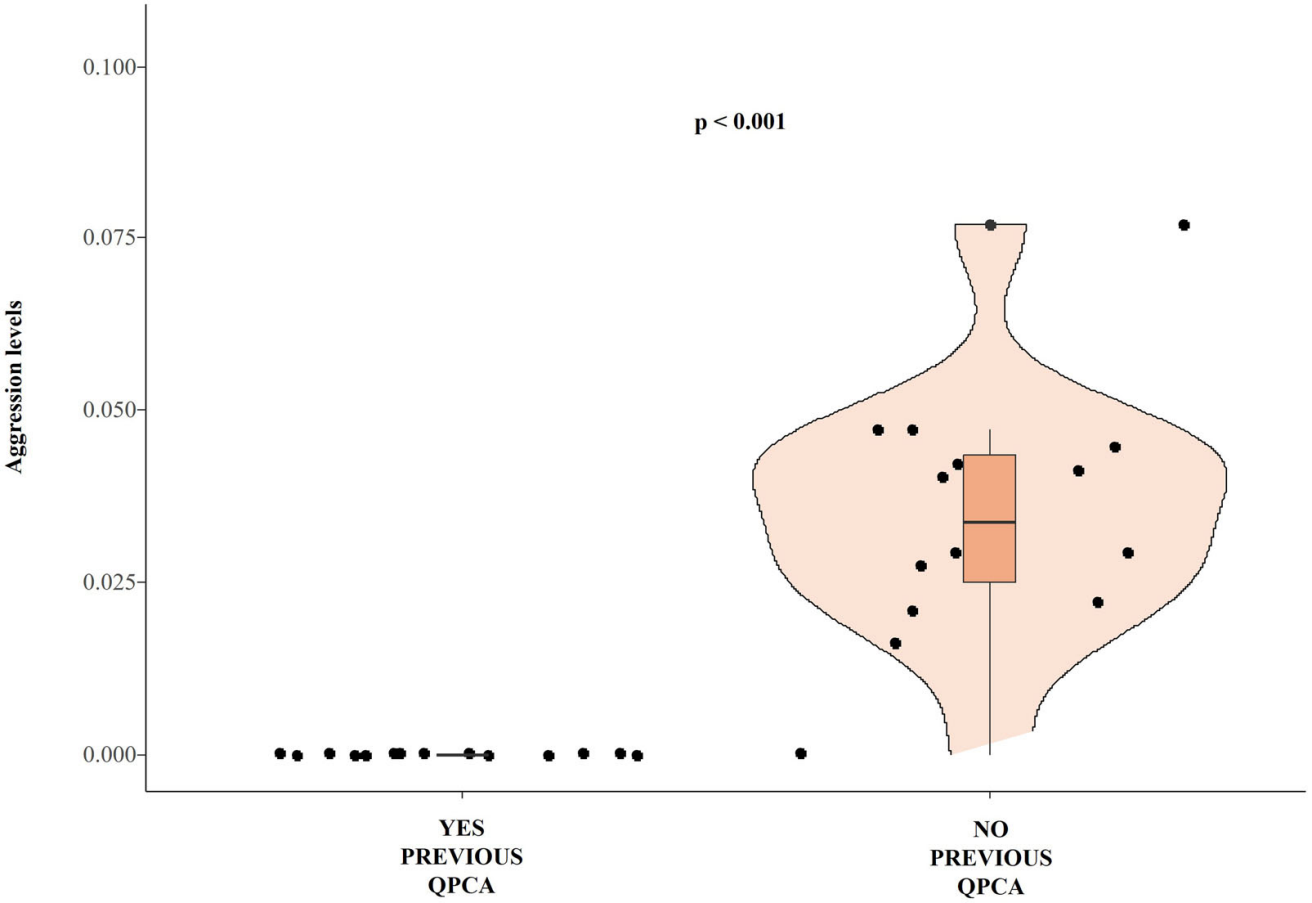
Violin plots with included boxplots representing aggression levels directed towards bystanders in the presence of previous quadratic post‐conflict affiliation (YES PREVIOUS QPCA) and in the absence of QPCA (NO PREVIOUS QPCA). The shape of the violin represents the density estimate of the variable considered: the more data points in a specific range, the larger the violin shape is for that range. Dots represent the individual data points (*N*_individuals_ = 15). In the middle of each density curve, there is a small boxplot with the rectangle showing the ends of the first and third quartiles and the central line showing the median value.

**Prediction *4*b**. The levels of aggression directed by group‐members towards bystanders were higher in the absence compared to the presence of quadratic affiliation (Wilcoxon exact test *N*_individuals_ = 15, *T* = 0, ties = 1, *p* < 0.001; median values and interquartile range: PC_NO_ 0.041/0.02; PC_YES_ 0.0/0.0; Figure 5).

Prediction 5 Relation between QPCA and other post‐conflict mechanisms

The sequential analysis showed that—when presented—quadratic affinitive contacts occurred significantly more frequent before rather than after reconciliation (Wilcoxon exact test Bonferroni's correction *α* = 0.01 *N*_individuals_ = 15, *T* = 0, ties = 1, *p* < 0.001), triadic unsolicited contacts with both aggressor (*N*_individuals_ = 15, *T* = 0, ties = 0, *p* < 0.001) and aggresse (*N*_individuals_ = 15, *T* = 0, ties = 1, *p* < 0.001), and triadic solicited contacts with both aggressor (*N*_individuals_ = 15, *T* = 0, ties = 0, *p* < 0.001) and aggresse (*N*_individuals_ = 15, *T* = 0, ties = 1, *p* < 0.001).

## 4 Discussion

In social groups, there are mechanisms that allow individuals to be affected by others' emotions and—under suitable subjective and contextual conditions—take action (e.g., Prochazkova and Kret 2017; Decety et al. 2016; de Waal and Preston 2017). After observing a stressful event, namely a conflict, social animals can engage in various post‐conflict affinitive interactions, including interactions with other bystanders (de Waal and van Roosmalen 1979; Judge and Mullen 2005; Cordoni et al. 2022). In the current study, we demonstrated for the first time in chimpanzees the occurrence of post‐conflict affiliation between bystanders who witnessed a conflict (i.e., quadratic post‐conflict affiliation – QPCA; *Prediction 1* supported). The mean group QPCA tendency is rather low (5.60% ± 2.55 SE). Certainly, the other post‐conflict mechanisms (reconciliation and solicited/unsolicited triadic contacts) play a pivotal role in managing the social uncertainty resulting from previous aggressive interactions, as they involve the main actors in the conflict—the opponents. However, bystanders, on the other hand, may need to manage the uncertainty related to the potential spread of aggression, which could also involve them. In this sense, QPCA plays an important role as discussed below. QPCA was directed more frequently towards males than females (*Prediction 2a* supported). Neither relationship quality nor kinship affected the levels of QPCA, but high‐ranking bystanders were contacted more frequently than low‐ranking ones (*Prediction 2b* partially supported). Neither individual and social factors linked to the original opponents, nor the aggression features influenced the occurrence of QPCA (Predictions 3a and 3b not supported). QPCA did not reduce the bystander anxiety‐related behaviors, at least within the chimpanzee group under study (Prediction 4a not supported). However, QPCA may serve as a protective tool for bystanders by limiting the probability of renewed aggression towards them, as it has been observed in Japanese macaques (Daniel and Alves 2015; Prediction 4b supported). Finally, QPCA occurred more frequently in absence than in presence of both reconciliation and triadic contacts (Prediction 5 supported).

In chimpanzees, males are the dominant sex, and they typically contest their rank through aggression, particularly towards other males (Muller 2002; Muller et al. 2021). Males may also direct aggression towards females for sexual coercion, dominance expression, or food competition (Kaburu and Newton‐Fisher 2015; Muller 2017; Enigk et al. 2021). For example, adolescent males (9–14 years old)—which were present in our group too (see Table 1)—can use aggressive behavior against females to increase their hierarchical position (Enigk et al. 2021). In line with this, our findings showed that males initiated aggression more frequently than females. Furthermore, high‐ranking males primarily directed their aggressive contacts towards low‐ranking females. In this context, it seems reasonable that QPCA was mainly directed towards males, as males could be most likely influenced by the ongoing aggression and may potentially direct further aggression towards bystanders. Thus, QPCA may function as a sort of “appeaser,” by calming down specific bystanders who may otherwise become potential aggressors. Accordingly, our findings showed that bystanders were less frequently targeted by aggression in the presence rather than in the absence of QPCA. The *Victim Protection Hypothesis* has been proposed in relation to the triadic post‐conflict contacts, both solicited and unsolicited (Palagi and Norscia 2013). According to this hypothesis, affinitive contacts may protect the aggressee from receiving further attacks by the previous aggressor or other group‐members. In our case, we may suggest a *Bystander Protection Hypothesis* based on the role of QPCA in reducing the risk of bystanders to be attacked. Further investigation is necessary to investigate whether the quadratic contacts actually reduce attacks toward the bystanders.

In the group under study, chimpanzee bystanders experienced more anxiety around aggression (PRE, PC_NO_, PC_YES_) than during baseline conditions. The same trend has also been observed in Japanese macaques (Daniel and Alves 2015), mandrills (Schino and Sciarretta 2015), and baboons (Judge and Mullen 2005). These results highlight once again that aggressive events affect the internal states of all group members, even those not directly involved in the conflict. In our chimpanzee group, QPCA did not appear to have an anxiolytic effect for bystanders, as it was observed in some cases for quadratic affiliation in other species (Judge and Mullen 2005; De Marco et al. 2010; Leone et al. 2010; Schino and Sciarretta 2015). Levels of anxiety‐related behaviors in bystanders did not significantly differ in the presence or absence of QPCA. This finding suggests that QPCA in chimpanzees may serve more as a protective than a calming tool for bystanders. In wild chimpanzees, even during resting lower‐ranking males showed higher levels of anxiety compared to higher‐ranking males, whereas females were more anxious when staying in proximity with non‐friends or non‐kin (Kutsukake 2003). This suggests that, even in low‐tension situations (e.g., resting) or following affinitive contact (as in the case of QPCA), individual anxiety levels remained quite elevated due to other social factors (e.g., interindividual dominance relationships). In this light, it is worth noting that in captivity, compared to the wild, interindividual distances were shorter. Consequently, after a conflict, individuals are ‘obliged’ to remain in close proximity to both previous opponents and other group members, which can increase the risk of further attacks. Furthermore, in this condition the uncertainty (Cords and Aureli 2000; Aureli and Schaffner 2002) over relationships may be higher and this condition may prevent individuals from predicting the reactions of conspecifics. In captive chimpanzees, Koski et al. (2007) found that reconciliation also did not reduce anxiety levels in the aggressee, contrary to expectations. Thus, post‐conflict behavioral mechanisms may not always have an anxiolytic effect in the very short‐term due to various individual (e.g., sex, age) and social (e.g., ranking position, relation quality) factors possibly affecting conflict management dynamics.

The opponent identities, the relationships between opponents and bystanders, and the features of aggression did not affect the presence of QPCA as observed, for instance, in mandrills (Schino and Sciarretta 2015). If bystanders were merely seeking contact with other conspecifics, their choice of partner would not necessarily depend on the identity of the opponents. This response might be considered a “reactive” quadratic interaction, as bystander behavior would likely be a reaction to the general agonistic context rather than to the specific individuals engaged in aggression (Judge and Mullen 2005). Furthermore, QPCA levels were not influenced by either relationship quality or kinship among bystanders. This finding contrasts with previous research on Tonkean and Japanese macaques (De Marco et al. 2010; Daniel and Alves 2015), two macaque species that exhibit tolerant and despotic social relationships, respectively (Aureli et al. 1992, 1993; Kutsukake and Castles 2001; Thierry 2000, 2007; Zannella et al. 2017). In chimpanzees, tolerance between group‐members varies selectively based on individual and environmental factors and can differ widely among groups (Brosnan et al. 2005; Bray et al. 2021; DeTroy et al. 2021; Fox et al. 2024). However, tolerance and affiliation (often used to assess relationship quality) represent different aspects of interindividual bonds (Lee et al. 2019; Dale et al. 2020; Schülke et al. 2022). Tolerance allows individuals to remain in proximity around valuable resource (e.g., food, estrus females) without aggression (DeTroy et al. 2021; Cronin and Sánchez 2012), and this can occur independently of the level of affiliation between individuals. Interestingly, our findings demonstrated that QPCA was primarily directed towards high‐ranking individuals. This evidence suggests that rank more than relationship quality may modulate QPCA, at least within the group under study. According to the *Current Needs Hypothesis* (Henzi and Barrett 2002, 2007; Seyfarth and Cheney 2012), exchanges between group members depend on individuals' current needs and the immediate availability of resources. In this sense, individuals make decisions based on what best serves their immediate interests (Henzi and Barrett 2002, 2007; Seyfarth and Cheney 2012). We may assume that in our group, low‐ranking bystanders would prefer to direct QPCA towards high‐ranking bystanders as the latter can potentially provide immediate protection against other aggressors and offer greater tolerance. Once more, this result goes in support of the bystander protection hypothesis formulated above. Certainly, this explanation does not exclude the possibility that QPCA may also serve to establish and strengthen social bonds between bystanders to gain future benefits such as food sharing (for low‐ranking individuals) or coalitionary support (for high‐ranking individuals). Further studies are necessary in this respect.

Finally, in our chimpanzees, QPCA occurred more frequently in the absence than in the presence of both reconciliation and triadic contacts (solicited and unsolicited). It has been demonstrated—both in the wild and in captivity—that in chimpanzees triadic unsolicited contacts (including consolation) occurred more frequently in the absence of reconciliation (Palagi et al. 2006). For this reason, consolation has been considered as a substitute or alternative for conciliatory contacts (Wittig and Boesch 2003; Palagi et al. 2006; Koski et al. 2007; Fraser et al. 2008). However, QPCA does not directly involve the opponents, unlike reconciliation and triadic contacts and in this regard, it may not serve as a substitute or alternative for either of these mechanisms (i.e., reconciliation and triadic contacts). We may hypothesize that QPCA primarily occurs when the original aggression has not been resolved, and probably when the risk of redirection or renewed aggression is high due to the elevated anxiety levels among all group members. Accordingly, in hamadryads, Judge and Bachmann (2013) demonstrated that arousal in bystanders increased after an aggressive interaction but decreased following reconciliation between the former opponents. However, according to Koski et al. (2007), we cannot exclude the possibility that the different post‐conflict mechanisms operate independently and that diverse individual, social, and contextual factors may affect the decision‐making process behind dyadic, triadic, and quadratic post‐conflict interactions.

### 4.1 Constraints on Generality (Simons et al. 2017) and Conclusion

Although our study focused on only one captive chimpanzee group, and caution is needed when generalizing these findings at the population level, this study demonstrates that the phenomenon of QPCA is present in a great ape species. Chimpanzees are affected by their social environment and strategically cope with social challenges. Aggression events are group affairs, and QPCA may be one of the behavioral tools used to preserve group cohesion and reduce aggression. Further studies are encouraged to clarify the issue regarding QPCA and anxiety‐reduction to provide a more complete understanding of conflict management strategies in chimpanzees as well as in other primate and non‐primate species.

## Author Contributions

**Giada Cordoni:** conceptualization, methodological approach, formal analysis, training, writing – original draft preparation, writing – review and editing. **Ivan Norscia:** methodological approach, training, writing – original draft preparation, writing – review and editing. **Annarita Perri:** data collection and curation, video analysis. **Andrea Pierdomenico:** data collection and curation. **Baptiste Mulot:** facilities and data collection facilitation.

## Supporting information

AJP Raw data PC MC pairs.

AJP Raw data QPCA anxiety‐related behaviors.

AJP Raw data QPCA before after reconciliation triadic contacts.

AJP Raw data QPCA sex rank.

AJP Raw data QPCA subsequent aggression.

## Data Availability

The data that supports the findings of this study are available as Supporting Information of this article.
